# Acute Promyelocytic Leukemia Presenting as Bilateral Acute Limb Ischemia and ST Elevation Myocardial Infarction: A Case Report

**DOI:** 10.7759/cureus.8495

**Published:** 2020-06-07

**Authors:** Miguel A Chavez, Behnam Heidari, Sameer Thacker, Leena L Samuel, Martina Ogbonna

**Affiliations:** 1 Internal Medicine, Houston Methodist Hospital, Houston, USA

**Keywords:** acute limb ischemia, acute promyelocytic leukemia, peripheral revascularization, coagulopathy, acute arterial thrombosis

## Abstract

Acute myelogenous leukemia (AML) is one of the most common hematologic malignancies. Among them, acute promyelocytic leukemia (APL) is well known for its coagulopathies. Bleeding secondary to disseminated intravascular coagulation, is a common initial presentation and carries a high risk for mortality if left untreated. Thrombotic complications are uncommon and can be related to treatment with chemotherapeutic agents. Large artery thrombosis is very rare, and standardized management remains elusive given the classic revascularization techniques carry a significant risk of re-thrombosis, as well as high risk for mortality given the multiple surgical and percutaneous interventions that are attempted. A multidisciplinary approach is necessary in these cases to carefully weigh the risk and benefits as the classical approach to revascularization and acute arterial thrombosis could potentially cause harm.

## Introduction

Acute myelogenous leukemia (AML) often presents with vague symptoms like fever, excessive fatigue, bleeding, body aches, dyspnea, hepatosplenomegaly, or lymphadenopathy [1]. Acute promyelocytic leukemia (APL) has bleeding as the common initial presentation. Thrombotic complications are often related chemotherapeutic agents and central venous catheters, particularly in the APL variant [2]. In rare cases, an ischemic phenomenon associated with APL is noted and poses a significant mortality risk to patients despite medical and surgical management [3]. Here we present a rare case of APL which initially presented with thrombosis to highlight the need for a multidisciplinary approach given the propensity for complications.

## Case presentation

A 71-year-old man presented to the ED with a two-day history of bilateral lower extremity pain. He initially experienced left lower extremity pain and cold sensation which progressed to the right lower extremity. Pertinent past medical history included type 2 diabetes mellitus, hypertension, coronary artery disease with percutaneous coronary intervention (PCI) to the right coronary artery (RCA) 18 years prior, peripheral arterial disease (PAD) with bilateral superficial femoral artery stenting two years prior and atrial fibrillation with recent ablation.

**Figure 1:**
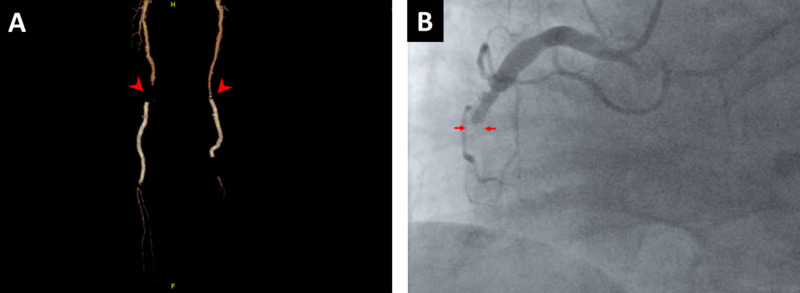
3D-Run off reconstruction from CT lower extremity angiogram (A) and selective left heart catheterization (B). A: Bilateral popliteal stents are shown, with bilateral proximal filling defect (red arrowheads) and poor distal reconstitution, indicating stenosis. B: Large ectatic RCA, with abrupt mid-RCA filling defect, diagnostic for 100% in stent thrombosis (red arrows). RCA, right coronary artery

Physical exam revealed cold lower limbs and there was bilateral absence of dorsalis pedis and posterior tibial pulses. He had decreased sensation to light touch with preserved strength. The remainder of the physical exam including heart and lung auscultation was unremarkable. Arterial Doppler studies showed occluded stent grafts in the superficial femoral arteries and popliteal arteries bilaterally. A subsequent CT angiogram of his lower extremities confirmed these findings, revealing minimal flow within the dorsal right calf arteries, with no flow being identified within the left calf arteries (Figure 1A). 

**Figure 2:**
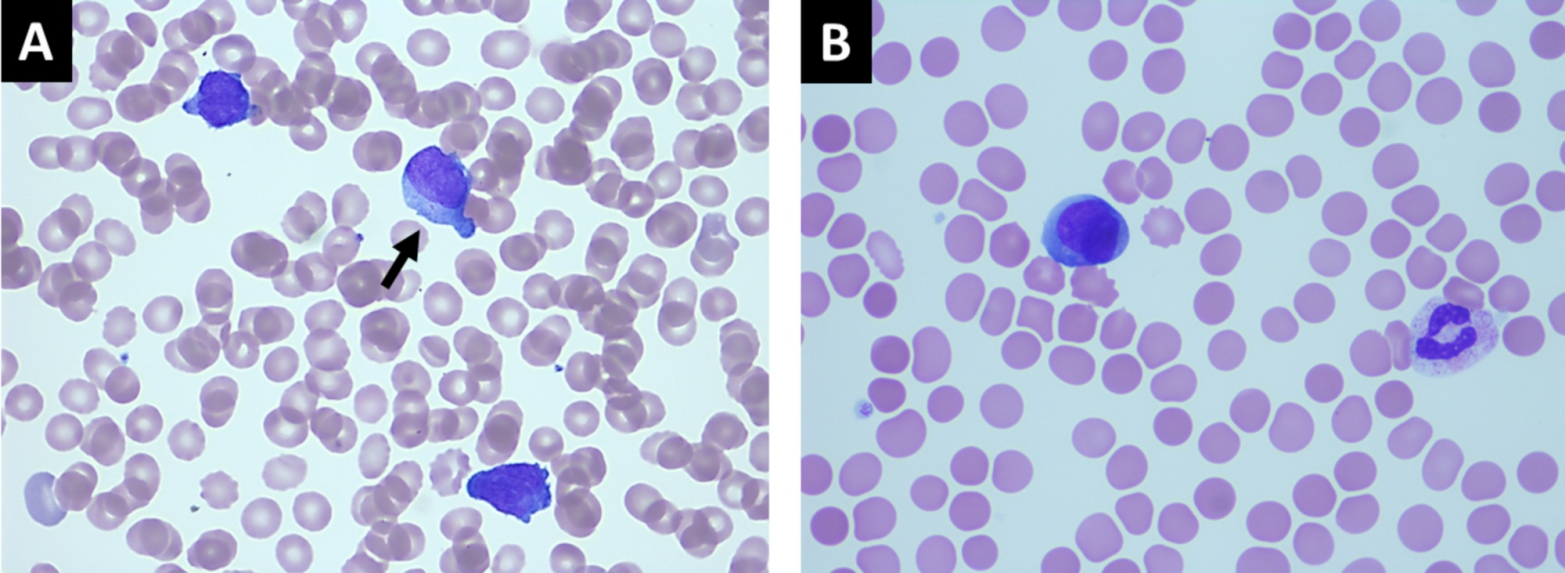
Peripheral blood smears at 1000x magnification using H&E stain showing circulating neoplastic promyelocytes. A: Three circulating promyelocytes with varying degrees of nuclear irregularity, one of which contains a small Auer rod (black arrow). B: Single promyelocyte with hypogranular cytoplasm and nuclear bilobation, typical of microgranular acute promyelocytic leukemia H&E stain: hematoxylin and eosin stain

**Figure 3:**
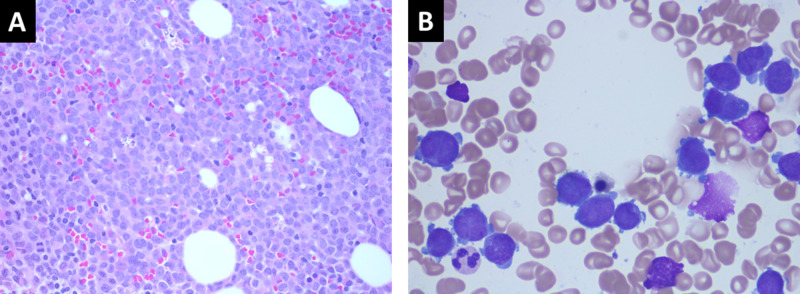
H&E stain of bone marrow core biopsy (A) and Wright-Giemsa stain bone marrow aspirate (B) at 400x and 1000x magnification respectively. A: Sheets of immature cells with eosinophilic cytoplasm and irregular nuclei with immature, fine chromatin and prominent nucleoli. B: Numerous predominantly hypogranular neoplastic promyelocytes, most of which show lobated nuclei with overlapping of nuclear lobes. H&E stain: hematoxylin and eosin stain

His initial laboratory results were remarkable for hemoglobin 12.4 g/dL, mean corpuscular volume 104.3, white blood cell (WBC) count 4.6 k/uL, and platelet count 46 k/uL. He was incidentally noted to have 30% peripheral blasts with features consistent with APL on peripheral smear review for thrombocytopenia which prompted a hematology consultation (Figure 2). Coagulation panel showed prothrombin time 17.9, international normalized ratio (INR) 1.5, D-dimer >20 ug/mL, and fibrinogen 86 mg/dL. He subsequently underwent bone marrow biopsy which revealed a t(15:17) promyelocytic leukemia/retinoic acid receptor alpha (PML-RARA) positive and 66% confirming diagnosis of APL (Figure 3). 

After a multidisciplinary discussion with vascular surgery and hematology, the patient was deemed to be very high risk for any surgical intervention and was continued on heparin drip. The following day, the patient reported numbness and weakness in his right lower extremity which resolved spontaneously, and the patient opted for continued observation and chemotherapy. Given the possibility of emergent intervention and concern for poor wound healing, the patient was started only on single agent all-trans retinoic acid (ATRA) alone instead of full-regimen chemotherapy.

His hospital course was complicated by atrial fibrillation with rapid ventricular response and subsequently developed ST elevation myocardial infarction (STEMI) needing emergency balloon angioplasty, subsequently sustained a cardiopulmonary arrest (Figure 1B). Despite a brief return of spontaneous circulation, the patient deteriorated rapidly and expired.

## Discussion

Bleeding is a relatively common presentation of APL-related coagulopathy, and is largely secondary to disseminated intravascular coagulation (DIC) and thrombocytopenia, but major thrombosis, especially arterial thrombosis, is very uncommon [4-5]. Because of its rarity, most data rely on case reports and other small retrospective studies.

In 2005, an observational cohort study of 379 patients at the Institute of Hematology in Rome sought to investigate the risk of thrombosis in AML [6]. Twenty-four patients (6.3%) had thrombosis, with only 20% being arterial in nature. At the time of diagnosis, thrombosis was the presenting symptom in 3.4% of the cases (1.4% acute lymphoblastic leukemia, 9.6% APL, 3.2% in non-M3 AML). After undergoing treatment, at six months follow up, the cumulative incidence of thrombosis was 10.6% in acute lymphoblastic leukemia (ALL), 8.4% in APL and 1.7% in non-M3 AML, suggesting that treatment may increase the risk of thrombosis, especially those who underwent treatment with L-asparaginase. Furthermore, Breccia et al. reported a prospective cohort of patients with APL who developed thrombosis while undergoing treatment with ATRA and idarubicin, describing a higher incidence of thrombosis in those patients with higher WBC count (mean 17 x 109), prevalence of break cluster region 3 (BCR3) transcript type, Fms related receptor tyrosine kinase 3-internal tandem duplication (FLT3-ITD), cluster of differentiation 2 (CD2), and cluster of differentiation 5 (CD5) expression [2]. Although our patient had FLT3-ITD mutation detected, he was found to have acute limb ischemia before any chemotherapeutic agent exposure, and did not have leukocytosis on presentation.

Over the past 30 years, there have been few cases of arterial thrombosis as a presenting phenomenon of APL. Involvement of large arterial vessels is very rare but have been described; these include the carotid artery or other intracranial vessels, iliac-femoral arteries, and even intra-cardiac thrombus [7-9]. These cases align with the findings in our patient, characterized by total bilateral superficial femoral artery occlusion, with concomitant development of complete mid-RCA occlusion during his hospitalization. Notably, the patient had evidence of peripheral vascular disease and coronary artery disease, as evidenced by his history of femoral-popliteal aneurysms and RCA stenting. Even though he had substantial cardiovascular disease, we argue that the nature of his arterial thrombosis was mostly driven by his underlying APL, given that he experienced simultaneous coronary and bilateral lower extremity thrombosis while being compliant with his anticoagulation and antiplatelet therapy. Late stent-thrombosis in PAD and coronary artery disease is not uncommon and has been described [10-11]. However, in our case, simultaneous thrombosis suggests a coagulopathy rather than stent-driven thrombosis; although one can argue that prior stenting could have increased his risk for this incident.

Currently, there is no high-quality evidence to standardize the management of acute limb ischemia in patients with active leukemia. In 2007, Kafetzakis et al. reported a case of acute limb ischemia as a presenting symptom of AML [12]. They summarized an additional nine cases with similar presentation that had been reported at that time. Eight (80%) patients underwent invasive management (e.g. thrombolysis, thrombectomy, bypass), with only one (10%) fatal outcome, however, four (40%) patients still required some sort of amputation. Two patients were solely managed with chemotherapy, with one of them requiring a minor amputation. More recently in 2016, a similar case was published by Pranit et al. and alluded to 10 additional cases [13]. Taking all of these cases into consideration, 15 out of 20 (75%) patients underwent invasive management with seven (45%) having a fatal outcome. In addition, three (20%) patients progressed to limb loss. Finally, eight (53%) patients who underwent invasive management had re-stenosis, requiring multiple attempts to revascularization, with some of them still requiring amputation.

Management of this case was particularly challenging, given the poor outcomes as reported in the previous studies. His initial presentation was highly concerning for acute limb ischemia and given his newly diagnosed APL, the patient was deemed a very high risk for re-thrombosis, by-pass graft failure, or bleeding after a procedure. He had findings of ongoing ischemia but did not exhibit any signs of an acute threatened limb and remained hemodynamically stable; therefore, a more conservative management with careful observation and initiation of IV heparin infusion was undertaken. Importantly, despite anticoagulation being the standard of care for venous thrombosis, limited evidence suggests that arterial thrombosis secondary to AML may not be as responsive to anticoagulation, as the mechanism of thrombosis may have a greater component of leukostasis with a higher composition of clot by leukemic cells and increased platelet aggregation secondary to WBC-induced vessel damage [12, 14-15]. The patient was also started on ATRA as his peripheral blasts began to increase despite a relatively stable total WBC count. Unfortunately, the patient became hemodynamically unstable after experiencing atrial fibrillation and STEMI, with rapid deterioration despite percutaneous balloon angioplasty and aggressive management in the ICU.

## Conclusions

Patients with APL presenting with acute limb ischemia secondary to thrombotic coagulopathy are rare but have been reported in the literature. A multidisciplinary approach must be undertaken to guide therapy, including careful evaluation of invasive management, as patients are at high risk for mortality, limb loss, or complications from such procedures. Optimal treatment remains unknown, as few cases have been reported, and treatment remains largely individualized.
